# Myeloid-specific *Hdac10* deletion protects against LPS-induced acute lung injury via P62 acetylation at lysine 165

**DOI:** 10.1186/s12931-024-02891-2

**Published:** 2024-07-02

**Authors:** Yuanyuan Xiang, Yu Zhong, Xianwen Lai, Zhenfu Fang, Guomei Su, Yingying Lv, Xiantong Tang, Lihuan Ouyang, Xiao Gao, Hongying Zheng, Lilin He, Jialong Chen, Jiewen Huang, Tianwen Lai

**Affiliations:** 1 Department of Respiratory and Critical Care Medicine, Dongguan Institute of Respiratory Medicine, The First Dongguan Affiliated Hospital, Guangdong Medical University, Dongguan, 523121 China; 2 Dongguan Key Laboratory of Immune Inflammation and Metabolism, Dongguan, 523121 China; 3 Institute of Respiratory Diseases, Affiliated Hospital of Guangdong Medical University, Zhanjiang, 524001 China; 4 The Department of Pulmonary and Critical Care Medicine, The Third People’ s Hospital of Xining City, Qinghai, 810005 China; 5 Department of Preventive Medicine, School of Public Health, Guangdong Medical University, Dongguan, 523121 China

## Abstract

**Background:**

Aberrant activation of macrophages is associated with pathogenesis of acute lung injury (ALI). However, the potential pathogenesis has not been explored.

**Objectives:**

We aimed to identify whether histone deacetylase (HDAC) 10 is involved in lipopolysaccharide (LPS)-exposed ALI and reveal the underlying pathogenesis by which it promotes lung inflammation in LPS-exposed ALI via modifying P62 with deacetylation.

**Methods:**

We constructed an ALI mice model stimulated with LPS to determine the positive effect of *Hdac10* deficiency. Moreover, we cultured murine alveolar macrophage cell line (MH-S cells) and primary bone marrow-derived macrophages (BMDMs) to explore the pro-inflammatory activity and mechanism of HDAC10 after LPS challenge.

**Results:**

HDAC10 expression was increased both in mice lung tissues and macrophage cell lines and promoted inflammatory cytokines production exposed to LPS. *Hdac10* deficiency inhibited autophagy and inflammatory response after LPS stimulation. In vivo, *Hdac10*^*fl/fl*^*-LysMCre* mice considerably attenuated lung inflammation and inflammatory cytokines release exposed to LPS. Mechanistically, HDAC10 interacts with P62 and mediates P62 deacetylation at lysine 165 (K165), by which it promotes P62 expression and increases inflammatory cytokines production. Importantly, we identified that Salvianolic acid B (SAB), an HDAC10 inhibitor, reduces lung inflammatory response in LPS-stimulated ALI.

**Conclusion:**

These results uncover a previously unknown role for HDAC10 in regulating P62 deacetylation and aggravating lung inflammation in LPS-induced ALI, implicating that targeting HDAC10 is an effective therapy for LPS-exposed ALI.

**Supplementary Information:**

The online version contains supplementary material available at 10.1186/s12931-024-02891-2.

## Introduction

Acute lung injury (ALI) is a clinical syndrome caused by a variety of direct or indirect factors [1]. Since the Berlin definition in 2012, ALI is commonly used to describe the general condition or animal models that fail to meet the clinical definition criteria [2]. Meanwhile, the potential mechanisms of ALI are not well understood, there is not yet an effective specific targeted therapy for ALI. Hence, there is an urgent need to investigate the pathogenesis and potential mechanisms underlying ALI.

Lung macrophages are essential for the inflammatory response in ALI. Under physiologic condition, alveolar macrophages (AMs) are involved in a variety of immune diseases and maintain airway homeostasis [3]. During the inflammatory period of ALI, AMs can produce various inflammatory cytokines [4]. It has been reported that adipose-derived mesenchymal stem cell-derived exosomes (AdMSC-Exos) can improve the integrity of mitochondrial components to promote macrophage homeostasis and alleviate inflammation in ALI [5]. Cluster of differentiation 36 (CD36) deficiency attenuates M1 polarization of macrophages and thus alleviates ALI [6]. Nevertheless, the role of autophagy in macrophages during LPS-stimulated ALI remains largely unexplored.

Acetylation, as a well-explored post-translational modification (PTM), intricately regulates various cellular physiological processes. Histone deacetylases (HDACs), crucial enzymes in this process, catalyze the removal of acetyl groups from histone lysines [7]. In recent studies, HDACs have been played an important role in ALI: HDAC5 deacetylates Lys136 of PP2Ac, inhibits its phosphatase activity, and negatively regulates the LPS-challenged ALI lung inflammation [8]. HDAC6 promotes the secretion of inflammatory cytokines in macrophages, activates the AP-1 and NF-κB pathways, and ultimately leads to ALI [9]. HDAC10 participated in temporomandibular arthritis, and HDAC10 overexpression activates the NF-κB pathway and aggravates inflammation response [10]. However, the mechanism of how HDAC10 regulates macrophage activation in ALI is still unclear.

Autophagy is a cellular mechanism that facilitates self-renewal and preservation by degrading proteins or its own organelles. Autophagy has gained significant attention due to its role in various diseases [11], including its significant role in ALI [12]. P62, a multidomain, multifunctional protein with 440 amino acids, involving in inflammation, cell homeostasis regulation, and cancer via various mechanisms by binding to various proteins [13]. Sitagliptin facilitates the activation of the p62-Keap1-Nrf2 pathway, thereby mitigating oxidative stress and hyperautophagy in ALI associated with severe acute pancreatitis [14]. Dapk1 suppresses autophagy and mitigates oxidative stress in LPS-treated ALI via inhibiting the p38MAPK/NF-κB signaling pathway [15]. These findings underscore the pivotal role of P62 in the inflammatory response characteristic of ALI. However, the underlying mechanisms by which it is involved remain unreported.

In our study, we observed an increase of HDAC10 expression in macrophages. Furthermore, our findings demonstrate that deficiency of HDAC10 confers protection against LPS-induced ALI. Mechanistically, HDAC10 interacts with P62, and HDAC10 acts as a regulator of P62^Lys165^ to promote lung inflammation of LPS-treated ALI.

## Results

### HDAC10 expression is increased in the lung macrophages following LPS exposure

**Fig. 1:**
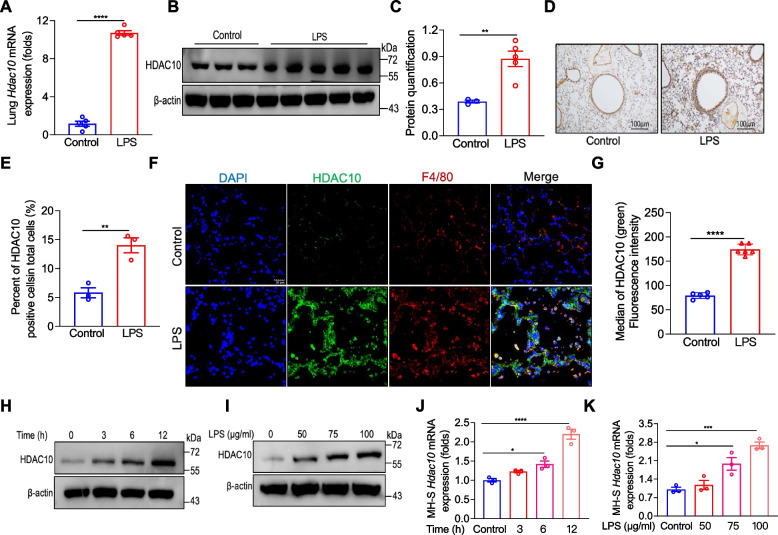
HDAC10 expression is increased in the lung macrophages following LPS exposure. **A** HDAC10 mRNA level in LPS-exposed ALI mice lung tissues. **B**, **C** HDAC10 protein level in LPS-stimulated ALI mice lung tissues. **D**, **E** Increased HDAC10 level in lung tissues of LPS-stimulated ALI mice measured by IHC staining for HDAC10. **F**, **G** IF staining of HDAC10 and F4/80 in mice lung tissues before or after LPS exposure. **H**, **I** WB analysis of HDAC10 in MH-S cells after 100 μg/ml LPS stimulation for 0-12 h and at different concentrations for 12 h. **J**, **K** HDAC10 mRNA expression in MH-S cells after 100 μg/ml LPS stimulation for 0-12 h and at different concentrations for 12 h. The data are expressed as the mean ±SEM (*n* = 3-5 mice/group). The data of Fig. 1G expressed as the median. ^*^p < 0.05, ^**^p < 0.01, ^***^p < 0.001, and ^****^p < 0.0001. Data are representative of three independent experiments with similar results (**A**, **E**, **J**, **K**). See also Fig. S1

To determine whether HDAC10 is involved in LPS-treated ALI, we initially detected HDAC10 expression in ALI mice lung tissues. Our findings revealed an obvious increase of HDAC10 mRNA levels in LPS-exposed ALI mice lung tissues compared to those of normal controls (Fig. 1A). These results were revealed by Western Blotting (WB) and immunohistochemistry (IHC) (Fig. 1B-E). Moreover, the immunofluorescent staining result showed that HDAC10 was co-localized with macrophage marker F4/80 (Fig. 1F, G). In addition, we detected HDAC10 expression in the MH-S cells exposed to LPS by WB, qRT-PCR (Fig. 1H-K) and immunofluorescence (IF) (Figure S1A, B). Then, we subjected BMDMs to similar LPS stimulation conditions as MH-S cells and observed HDAC10 expression was increased after LPS stimulation (Figure S1C-F). Collectively, these results indicated that HDAC10 was obviously expressed after treated with LPS in macrophages, which encouraged us to further investigate its role in LPS-induced ALI.

### *Hdac10* deficiency attenuates LPS-induced lung inflammation in ALI

**Fig. 2:**
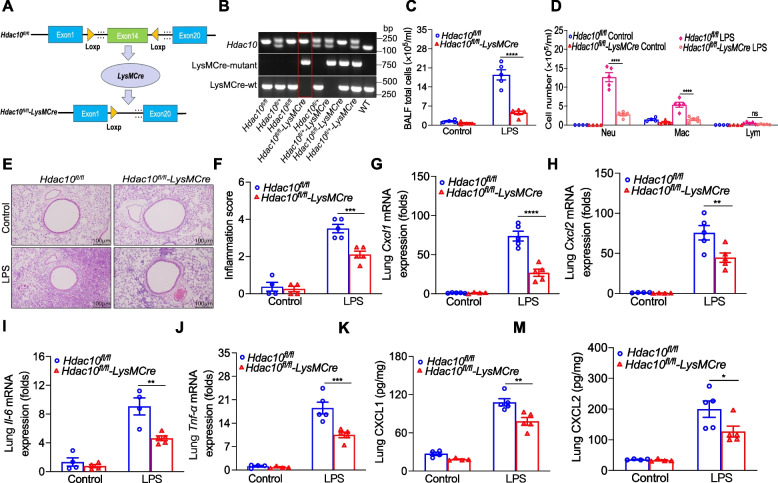
*Hdac10* deficiency attenuates LPS-induced lung inflammation in ALI. **A** Schematic diagram of *Hdac10*^*fl/fl*^*-LysMCre* mice. **B** Genotyping assessed by qRT-PCR using mouse tail genomic DNA. **C**, **D** Total cells and differential cells counts in BALF. **E**, **F** Histological analyses and inflammation score of mice lung tissues. **G**-**J** The mRNA levels of *Cxcl1*, *Cxcl2*, *Il-6* and *Tnf-α* in mice lung tissues. **K**, **M** Protein levels of CXCL1 and CXCL2 in mice lung tissues measured by ELISA. The data are presents as the mean ± SEM and represent three independent experiments (**C**,** D**,** F**-**M**). ^*^p < 0.05, ^**^p < 0.01, ^***^p < 0.001, and ^****^p < 0.0001. See also Fig. S2

To explore how HDAC10 regulates lung inflammation of ALI, we constructed *Hdac10*-deficient (mainly in macrophages) mice using genetic engineering (Fig. 2A). The mice representative genotyping results as shown in Fig. 2B. Then, Fig. S2A, B results showed that HDAC10 gene was successfully knocked out. The *Hdac10*^*fl/fl*^*-LysMCre* and *Hdac10*^*fl/fl*^ mice were stimulated with LPS according to the following method. The total cells of mice bronchoalveolar lavage fluid (BALF) in *Hdac10*^*fl/fl*^*-LysMCre* mice were dramatically lower than those in *Hdac10*^*fl/fl*^ mice treated with LPS (Fig. 2C, D). Hematoxylin & eosin (HE) staining was decreased in LPS-induced *Hdac10*^*fl/fl*^*-LysMCre* mice (Fig. 2E, F).

The secretion of inflammatory cytokine in *Hdac10*^*fl/fl*^*-LysMCre* mice lung tissues exhibited a significant reduction compared to that observed in *Hdac10*^*fl/fl*^ mice treated with LPS (Fig. 2G-J). Then, we also detected the protein levels of CXCL1 and CXCL2, the results showed by ELISA (Fig. 2K, M). We further cultured BMDMs isolated from *Hdac10*^*fl/fl*^ and *Hdac10*^*fl/fl*^*-LysMCre* mice, which were exposed to LPS for 3 h, and found that *Hdac10* knockout reduced inflammatory cytokine production (Fig. S2C-F). Collectively, these datas suggested that *Hdac10* deficiency in macrophages was sufficient to repress LPS-exposed lung inflammation.

### *Hdac10* deficiency suppresses autophagy to inhibit inflammatory cytokine production

**Fig. 3:**
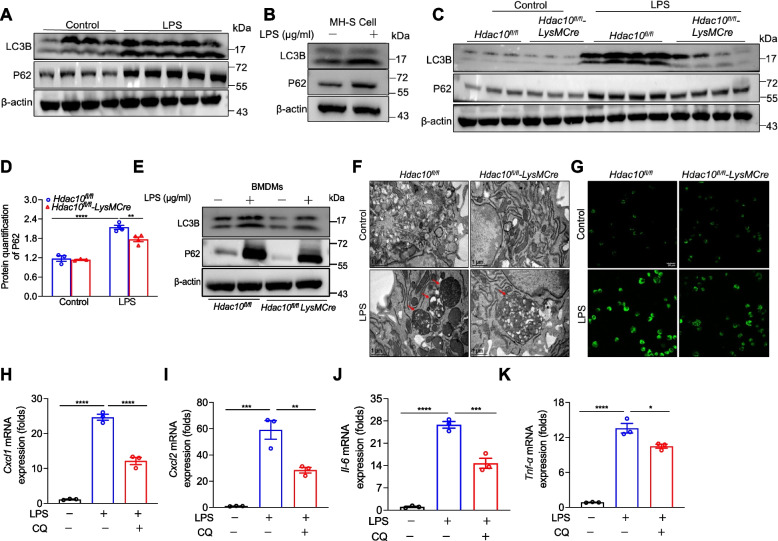
*Hdac10* deficiency suppresses autophagy to inhibit inflammatory cytokine production. **A** LC3B and P62 protein expressions in mice lung tissues. **B** LC3B and P62 protein levels in MH-S cells after LPS stimulation for 6h. **C** WB analysis of LC3B and P62 in lung tissues of mice. **D** Protein quantification of P62. E LC3B and P62 protein expressions in BMDMs with or without LPS stimulation for 6h. **F** Changes of the autophagosomes in BMDMs with or without LPS stimulation (100 μg/ml 2h) were analyzed by transmission electron microscope. **G** Changes of the autophagosomes in BMDMs exposed to LPS (100 μg/ml 2h) showed by fluorescence microscope. **H-K **The mRNA levels of *Cxcl1*, *Cxcl2*, *Il-6*, and *Tnf-α* in MH-S cells following LPS (100 μg/ml 3h) and CQ (10 μmol/ml 6h) exposure. The data are presents as the mean ± SEM and represent three independent experiments (**D**, **H**-**K**). ^*^p < 0.05, ^**^p < 0.01, ^***^p < 0.001, and ^****^p < 0.0001

Autophagy plays a key role in ALI. P62, a selective receptor of autophagy, regulates cell survival, inflammation and participates in a number of diseases [13]. However, it is unknown whether P62 participates in LPS-induced ALI and whether HDAC10 regulates P62 in macrophages. To confirm this question, we first assessed the expressions of LC3B and P62. We discovered that LC3B and P62 expressions were increased in lung tissues of ALI mice and LPS-treated MH-S cells (Fig. 3A, B), whereas the expressions of LC3B and P62 in the lung tissue of *Hdac10*^*fl/fl*^*-LysMCre* ALI mice were lower than those of *Hdac10*^*fl/fl*^ ALI mice (Fig. 3C, D). Consistently, the expressions of LC3B and P62 were reduced in *Hdac10*^*fl/fl*^*-LysMCre* BMDMs compared with *Hdac10*^*fl/fl*^ BMDMs after LPS challenge (Fig. 3E). Moreover, the inhibition of autophagy in *Hdac10*^*fl/fl*^*-LysMCre* BMDMs exposed to LPS was assessed by transmission electron microscopy (TEM) and Monodansylcadaverine (MDC) staining (Fig. 3F, G). Then, we found that autophagy inhibitor-chloroquine (CQ) treatment reduced inflammatory cytokines production in MH-S cells after exposure to LPS (Fig. 3H-K). In conclusion, inhibition of autophagy alleviated the inflammation of ALI. Therefore, our findings discovered that HDAC10 regulates the inflammatory response of LPS-exposed ALI by affecting autophagy, and *Hdac10* deficiency suppresses autophagy to inhibit inflammatory cytokine production.

### HDAC10 directly binds with P62 and targets P62 for deacetylation

**Fig. 4:**
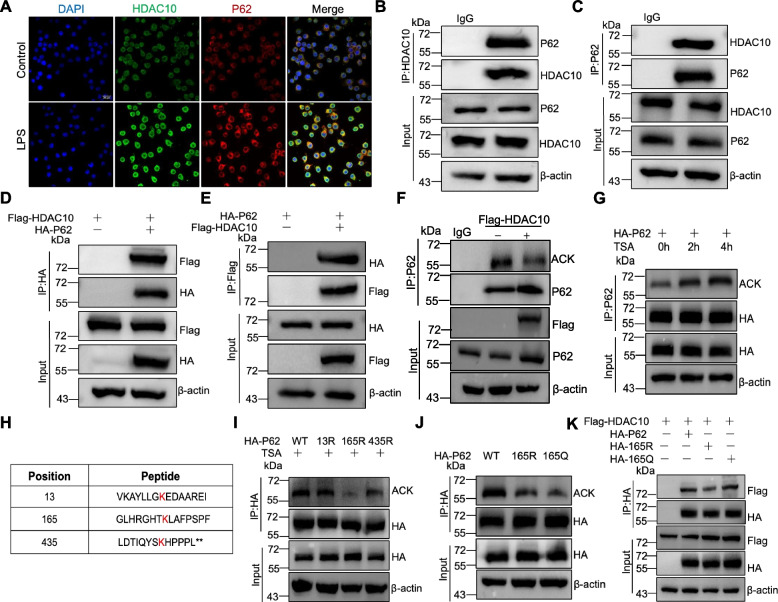
HDAC10 directly binds with P62 and targets P62 for deacetylation. **A** The colocalization of HDAC10 (green) and P62 (red) in MH-S cells were showed by immuno-fluorescence staining. **B**, **C** Endogenous HDAC10 or P62 in MH-S cells was evaluated by Immunoblot (IB). **D**, **E** IB analysis of exogenous HDAC10 or P62 in HEK 293T cells transfected alone or in combination with tagged HDAC10 and evaluated with Flag-HDAC10 and HA-P62 before (input) or after IP. **F** HEK 293T cells were transfected with labeled HDAC10, and the acetylation level of P62 was measured by IP. **G** Acetylation of exogenous HA-P62 in HEK 293T cells exposed to the deacetylase inhibitor trichostatin A (TSA), and the acetylation level of P62 was measured by IP. **H** Identification of acetylated P62 peptides by using CSS-Palm 3.0. **I** HEK 293T cells were transfected with HA-P62 (WT), HA-K13R, HA-K165R and HA-K435R for 48 h, and the acetylation levels of HA-P62 (WT), HA-K13R, HA-K165R and HA-K435R were measured by IP. **J** HEK 293T cells were transfected with HA-P62 (WT), HA-K165R and HA-K165Q for 48 h, and the acetylation levels of HA-P62 (WT), HA-K165R and HA-K165Q were measured by IP. **K** IB analysis of exogenous HDAC10, P62, K165R and K165Q in HEK 293T cells transfected alone or in combination with tagged HDAC10 and evaluated with Flag-HDAC10 and HA-P62 (WT), HA-K165R and HA-K165Q before (input) or after IP

To explore the mechanisms of HDAC10 regulating P62 during ALI, we have done some experiments. Coimmunostaining confirmed the expression and colocalized of HDAC10 and P62 in MH-S cells (Fig. 4A), while co-immunoprecipitation (Co-IP) determined interaction between HDAC10 and P62. These results demonstrate the coimmunoprecipitation of HDAC10 and P62, indicating their potential interaction in cellular processes (Fig. 4B-E).

We have confirmed that HDAC10 interacts with P62, and HDAC10 is a deacetylase that potentially mediates the deacetylation of P62. We found that the acetylation level of P62 was decreased after HDAC10 overexpression in HEK 293T cells (Fig. 4F). Then, we overexpressed P62 in HEK 293T cells, which was stimulated by the histone deacetylase inhibitor trichostatin A (TSA). We found that P62 acetylation was increased with the extension of TSA stimulation time (0 h~4 h) (Fig. 4G). To determine potential acetylated lysine site of P62, Acetylated lysine (K) sites of P62 were further predicted using CSS-Palm 3.0. and three acetylated lysine sites (K13, K165 and K435) were performed (Fig. 4H). Then, we constructed acetylation defective P62 mutants with each lysine residue replaced by arginine (K13R, K165R, and K435R) and detected their deacetylation. Notably, the K165R mutant, but not K13R or K435R mutants, exhibited reduced acetylation levels (Fig. 4I). K165 after simulated acetylation (Q) and deacetylation (R), their acetylation levels were detected by IP, the acetylation levels of WT-P62 were increased, and the acetylation levels of K165R and K165Q were decreased (Fig. 4J). In addition, we co-overexpressed Flag-HDAC10, HA-P62 (WT), HA-K165R, and HA-K165Q in HEK 293T cells, and evaluated the interaction between HDAC10 and P62, K165R, and K165Q via Co-IP. The results indicated a decreased interaction between HDAC10 and K165R compared to that between HDAC10 and P62 and K165Q (Fig. 4K).

### HDAC10 deacetylates P62-K165 to regulate inflammation in LPS-exposed ALI

**Fig. 5:**
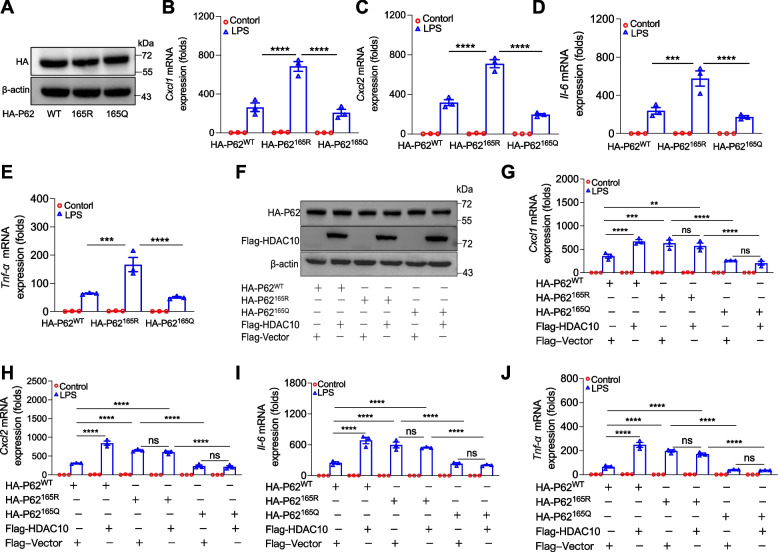
HDAC10 deacetylates P62-K165 to regulate inflammation in LPS-exposed ALI. **A** The transiently transfection efficiency of WT-P62 or P62 mutant plasmids (K165R and K165Q) in *Hdac10*^*fl/fl*^ BMDMs. **B**-**E** *Hdac10*^*fl/fl*^ BMDMs transiently transfected with WT-P62 or P62 mutant plasmids (K165R and K165Q) for 48h, and then BMDMs were stimulated with LPS for 3 h. The mRNA levels of *Cxcl1, Cxcl2, Il-6* and *Tnf-α* detected by qRT-PCR. **F** The transiently transfection efficiency of HDAC10, Vector, WT-P62 or P62 mutant plasmids (K165R and K165Q) in *Hdac10*^*fl/fl*^ BMDMs. **G**-**J** *Hdac10*^*fl/fl*^ BMDMs transiently transfected with HDAC10, Vector, WT-P62 or P62 mutant plasmids (K165R and K165Q) and then stimulated with LPS for 3 h. The mRNA levels of *Cxcl1, Cxcl2, Il-6* and *Tnf-α* in cells. The data are presents as the mean ± SEM and represent three independent experiments(**B-E**, **G**-**J**). ^*^p < 0.05, ^**^p < 0.01, ^***^p < 0.001, and ^****^p < 0.0001

We have confirmed that K165 was the major acetylation site of P62. Acetylation-defective substitution at K165 (K165R) significantly increased LPS-induced inflammatory cytokines production in *Hdac10*^*fl/fl*^ BMDMs, but K165Q (the lysine mutation to glutamine) had no such effect (Fig. 5A-E). Consistent with these findings, co-overexpression of HDAC10 and K165 prompted inflammatory cytokines secretion in *Hdac10*^*fl/fl*^ BMDMs (Fig. 5F-J). Furthermore, we investigated whether the elevation of P62-K165R could reverse the marked reduction in cytokine levels observed in *Hdac10*-deficient macrophages. The results showed that an increase in P62-K165R indeed counteracted the substantial decline in cytokine levels observed in *Hdac10*^*fl/fl*^*-LysMCre* BMDMs (Fig. S3A-E). Taken together, our datas suggested that HDAC10 regulates inflammation in LPS-induced ALI via deacetylating P62 at K165.

### HDAC10 inhibitor treatment attenuates LPS-induced lung inflammation in ALI

**Fig. 6:**
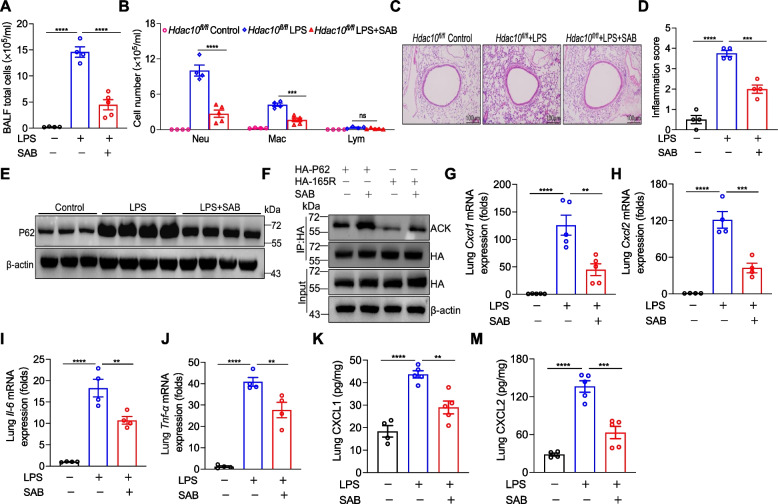
HDAC10 inhibitor treatment attenuates LPS-induced lung inflammation in ALI. **A**, **B** Total cells and differential cells counts of BALF. **C**, **D** Lung tissue sections stained with HE staining and inflammation score. **E** The protein level of P62 in mice lung tissues. **F** HEK 293T cells transiently transfected with WT-P62 or P62 mutant plasmids (K165R), and then HEK 293T cells were stimulated with SAB for 24 h. The acetylation levels of WT-P62 and K165R deteted by WB. **G**-**J** The mRNA levels of *Cxcl1*, *Cxcl2*, *Il-6* and *Tnf-α* in mice lung tissues. **K**, **M** The expressions of CXCL1 and CXCL2 of mice lung tissues detected by ELISA. The data are presents as the mean ± SEM and represent three independent experiments (**A**,** B**,** D**, **G**-**M**). ^**^p < 0.01, ^***^p < 0.001, and ^****^p < 0.0001. See also Fig. S3

Our findings discovered that targeting HDAC10 in LPS-induced ALI may be a potential treatment. We then evaluated the role of HDAC10 inhibitor Salvianolic acid B (SAB) in experimental mouse models. We observed a reduction in total cells of BALF in LPS-treated ALI mice following exposure to SAB (Fig. 6A, B). Additionally, HE staining revealed that SAB treatment mitigated lung inflammation in LPS-exposed mice (Fig. 6C, D). In addition, P62 protein level in LPS+SAB-treated mice was lower than LPS-exposed mice (Fig. 6E). To explore the effect of SAB on protein acetylation levels, we overexpressed P62 and K165R in HEK 293T cells and found that SAB increased the acetylation of both the whole protein and K165R (Fig. 6F). Moreover, the secretion of inflammatory cytokines in LPS+SAB-treated mice was decreased compared to those in LPS-exposed mice (Fig. 6G-J). We have also detected the protein levels of CXCL1 and CXCL2 by ELISA (Fig. 6K, M). Consistent with the in vivo findings, LPS-exposed MH-S cells exhibited elevated expression of P62 and inflammatory cytokines, which was reduced by SAB treatment (Figure S4A-E). To sum up, our findings hinted that the HDAC10 inhibitor SAB may play a significant role in reducing lung inflammation of LPS-treated ALI.

## Discussion

Our study suggested that HDAC10 was elevated in LPS-treated ALI mice and in vitro following LPS exposure. Mice with a macrophage-specific deletion of *Hdac10* alleviated lung inflammation in LPS-stimulated ALI. HDAC10 interacted with P62 and deacetylated P62 at lysine 165, by which facilitated the LPS-exposed ALI pulmonary inflammation. In additional, we found that SAB reduced the inflammatory response in LPS-stimulated ALI mice. Overall, our study discovered that HDAC10 played a crucial role in regulating lung inflammation by deacetylating P62^Lys165^, thus promoting lung inflammation in LPS-exposed ALI (Figure S5). ALI has a high morbidity and mortality in clinical practice [16], causing a huge economic burden to the society, if not treated in time, the patient will eventually die due to respiratory failure. Therefore, it is imperative to develop new strategies to target the molecular mechanisms of ALI. In our study, HDAC10 clearly exists as an adverse influence factor of ALI and determines the mechanism by which HDAC10 aggravates the lung inflammation of ALI.

As a key mechanism of epigenetics, histone modification involves alterations to chromatin structure through modifications of histones, thereby influencing gene expression. Consequently, HDACs are implicated in the pathogenesis of many diseases, such as inflammatory conditions, cancer, and immune disease [17]. HDAC10 stands out as a novel histone deacetylase, it deacetylates histones and inhibits transcription, and it plays a critical role in cell cycle development, hormone regulation, lipid metabolism and autophagy regulation [18]. It has been reported that HDAC10 deficiency enhances Foxp3+ T-regulatory cell function, and the HDAC10-specific inhibitors hold promise for treating immune-related disorders [19]. In addition, HDAC10 negatively regulates lung cancer development by promoting protein kinase B (AKT) phosphorylation [20]. These studies had shown that HDAC10 was particularly important in cell and lung development. However, there is no study to explore the role of HDAC10 in LPS-treated ALI. Here, we found that HDAC10 is significantly increased in mice lung tissues and cell lines exposed to LPS. *Hdac10* deficiency attenuates LPS-induced lung inflammation in ALI. In addition, we found SAB as a HDAC10 inhibitor that prevented lung injury and inflammation after LPS stimulation.

In recent years, more and more studies have shown that autophagy clears invading pathogens and involves in ALI/ARDS. Besides, the regulatory influence of acetylation on autophagy has attracted increasing attention. For instance, General Control Non-Repressed Protein 5 (GCN5) has been shown to acetylate transcription factor EB (TFEB), thereby reducing TFEB's transcriptional activation and subsequently decreasing lysosome formation during autophagy [21]. Furthermore, HDAC10 has been observed to interact with Microtubule-Associated Protein Light Chain 3 (LC3), an interaction inhibited by the autophagy inhibitor chloroquine. This inhibition promotes the phosphorylation of interferon regulatory factor 3 (IRF3), thereby enhancing IRF3-mediated antiviral immune responses [22]. Selective autophagy mediated by P62 has been implicated in the inflammation observed in sepsis, with autophagy deficiency being a significant contributing factor to sepsis development. It is worth noting that sepsis is one of the causes of ALI/ARDS. Li et al. found that Heme oxygenase-1 (HO-1) mitigated the inflammatory response in sepsis-induced ALI by activating the autophagy flux axis and inhibiting the NLRP3 inflammasome [23]. Although P62-mediated autophagy is implicated in ALI, its exact molecular mechanism remains unclear. Here, *Hdac10* deficiency significantly attenuated P62 expression in BMDMs following LPS exposure. We found that HDAC10 interacted with P62 and mediates P62 deacetylation at lysine 165. However, the underlying mechanisms of P62 deacetylation that regulates macrophage autophagy requires further investigation.

In summary, our experiments demonstrated that HDAC10 interacts with P62 and mediates P62 deacetylation at lysine 165 (K165), by which it promotes P62 expression and increases LPS-induced lung inflammation and may suggest that inhibition of HDAC10 could prevent the development of ALI-induced lung inflammation.

## Materials and methods

### Animal experiment

The *LysMCre* mice obtained from Dr. G. Feng (University of California, San Diego, USA). *Hdac10*^*fl/fl*^ mice were generated by Cyagen Biosciences Inc. (Suzhou, China) using the CRISPR-Cas9 system. To generate *Hdac10*^*fl/fl*^-*LysMCre* mice, the *LysMCre* mice were crossed with *Hdac10*^*fl/fl*^ mice to obtain it. The study protocol was approved by the Animal Ethics Committee of Guangdong Medical University.

### Acute lung injury mouse model

*Hdac10*^*fl/fl*^ and *Hdac10*^*fl/fl*^*-LysMCre* mice were used to construct acute lung injury (ALI) model, which were divided into *Hdac10*^*fl/fl*^ and *Hdac10*^*fl/fl*^*-LysMCre* control group, *Hdac10*^*fl/fl*^ and *Hdac10*^*fl/fl*^*-LysMCre* ALI group. To induce the ALI model, the mice were intratracheal instillated with 100 μg LPS in 50 μl normal saline. The HDAC10 inhibitor (salvianolic acid B) was intraperitoneally given to the mice 1 h before the LPS treatment. After 24 h of LPS stimulation, the mice were sacrificed by intraperitoneal injection of pentobarbital sodium (250 mg/kg).

### Histological and immunohistochemical analysis

Hematoxylin and eosin (HE) staining was performed to evaluate lung inflammation in ALI mice. Immunostaining staining was displayed co-location of HDAC10 and F4/80 in the mice lung tissues. Immunoconfocal imaging was performed on the sections.

### Quantitative RT-PCR (qRT-PCR)

Mice lung tissues and cell total RNA was extracted using Trizol reagent (Invitrogen, Carlsbad, CA, USA). Reverse transcription into cDNA using PrimeScript™RT kit (Takara, Beijing, China). The primer sequences were displayed in Table 1.

**Table 1:** Primers used for quantitative real time PCR analysis

Species/genes	Primer sequence
m-Hdac10 forward	5ʹ-ACAGCCACTCGACTGCTCT-3ʹ
m-Hdac10 reverse	5ʹ- GATGCCTCACAAGCTGACAAA -3ʹ
m-Cxcl-1forward	5ʹ-CTGGGATTCACCTCAAGAACATC-3ʹ
m-Cxcl-1 reverse	5ʹ-CAGGGTCAAGGCAAGCCTC-3ʹ
m-Cxcl-2 forward	5ʹ-TGTCCCTCAACGGAAGAACC-3ʹ
m-Cxcl-2 reverse	5ʹ-CTCAGACAGCGAGGCACATC-3ʹ
m-actin forward	5ʹ-AGTGTGACGTTGACATCCGT-3′
m-actin forward	5ʹ-GCAGCTCAGTAACAGTCCGC-3ʹ
m-Il-6 forward	5ʹ -TAGTCCTTCCTACCCCAATTTCC -3ʹ
m-Il-6 reverse	5ʹ -TTGGTCCTTAGCCACTCCTTC-3ʹ
m-Tnf-α forward	5ʹ -TAGCCCACGTCGTAGCAAAC-3ʹ
m-Tnf-α reverse	5ʹ -ACCCTGAGCCATAATCCCCT-3ʹ
h-Cxcl-1forward	5ʹ -AAGAACATCCAAAGTGTGAACG-3ʹ
h-Cxcl-1 reverse	5ʹ-CACTGTTCAGCATCTTTTCGAT-3ʹ
h-Cxcl-2forward	5ʹ -GCCACCACCTATTAGCCATGTCAC-3ʹ
h-Cxcl-2 reverse	5ʹ -GCTCTTTGTCATACTGGGCCTCAC-3ʹ
h-Il-6 forward	5ʹ -GCCCAGCTATGAACTCCTTCT-3ʹ
h-Il-6 reverse	5ʹ -CTTCTCCTGGGGGTACTGG-3ʹ
h-Tnf-α forward	5ʹ -CCCCAGGGACCTCTCTCTAA-3ʹ
h-Tnf-α reverse	5ʹ -GCTTGAGGGTTTGCTACAACA-3ʹ
h-actin forward	5ʹ -CCA TCA TGA AGT GTG ACG TGG-3ʹ
h-actin reverse	5ʹ -GTC CGC CTA GAA GCA TTT GCG-3ʹ

*m* mouse, *h* human

### Culture and treatment of BMDMs

BMDMs were isolated from C57BL/6J mice and were incubated in DMEM containing 10 ng/ml M-CSF (Novoprotein, Catalog #0331488), 10%FBS and 1% penicillin/streptomycin. BMDMs were exposed to LPS for 2h, 3h or 12h.

### Western blotting (WB)

Total proteins were lysed in RIPA buffer. After lysates were electrophoresed and blotted onto polyvinylidene fluoride membrane, which were finally incubated with the corresponding antibodies.

### ELISA

The concentrations of CXCL1 and CXCL2 in the mice lung tissues were measured using ELISA kits (eBioscience, Inc) following the instructions.

### Immunofluorescence (IF)

After cell samples fixed with 4% paraformaldehyde, they were incubated with the antibody against HDAC10 (1:150, Affinity, #AF5474) and P62 (1:150, Abcam, ab56416) overnight at 4 °C. Finally, cell samples were assessed by confocal microscopy (Olympus FV3000; Japan).

### Co-immunoprecipitation (Co-IP)

Protein samples were quantified using the BCA kit (Beyotime) and divided into input and IP groups. The IP group was incubated with 1 μg antibodies and 50 μl Protein G Beads overnight at 4 °C, while the input group was treated with 5×SDS Sample Buffer and boiled. After washing with IP lysis buffer (containing 1% PMSF), the IP group was boiled again with 2×SDS Sample Buffer.

### Transmission *electron* microscopy

BMDMs were cultured in 24-well plates containing cell culture plates and differentiated into macrophages stimulated with M-CSF. After stimulation with LPS for 3 hours, BMDMs were fixed in 2.5% glutaraldehyde, dehydrated, and embedded in epoxy resin. Samples were meticulously sectioned into ultrathin slices measuring 60-80 nm in thickness. Finally, autophagosomes were captured under transmission electron microscopy.

### Monodansylcadaverine (MDC) staining

Following stimulation with LPS for 3 hours, BMDMs were stained with MDC (50 µmol/L) for 30 min. Subsequently, BMDMs were washed with 1ml Assay Buffer for 3 times. Finally, the autophagy of cells was photoed by fluorescence microscope.

### Reagents and antibodies

Please see the Table 2.

**Table 2:** Key resources

REAGENT or RESOURCE	SOURCE	IDENTIFIER	
**Antibodies**			
Anti-β-actin	Beyotime	Cat#AA128	
Anti-P62	Abcam	Cab#ab56416	
Anti-LC3B	Sigma	Cab#L7543	
Anti-HDAC10	Santa Cruz	Cat#sc-393417	
Anti-HDAC10	Affinity	Cab#AF5474	
Anti-Flag	GenScript Biotech, china	Cab#A00187	
Anti-HA	Abbkine	Cat#ABT2040	
Anti-Acetyl Lysine	Immunechem	Cat#ICP0380	
Alexa Fluor 488	Beyotime	Cat#A0423	
Alexa Flour 555	Beyotime	Cat#A0460	
Anti-F4/80	Proteintech	Cat#29414-1-AP	
**Chemicals**			
Salvianolic acid B (SAB)	SparkJade	Cat#SJ-MN0081	
LPS	SIGMA	Cat#L2880-25MG	
TSA	Beyotime	Cat#P1112	
DAPI	Beyotime	Cat#C1005	
Protein A+G Agarose	Beyotime	Cat#P2055-50ml	
Recombinant mouse M-CSF	Novoprotein	Cat#CB34	
**Critical commercial assays**			
Mouse GROα/CXCL1 ELISA Kit	Elabscience	Cat#E-EL-M0018c	
Mouse GROβ/CXCL2 ELISA Kit	Elabscience	Cat#E-EL-M0019c	
TB Green® Premix Ex Taq™	Takara	Cat#RR420A	
Prime Script™ RT reagent Kit	Takara	Cat#RR047A	
Express Cast PAGE Gel Preparation kit	NCM Biotech	Cat#P2012	
MDC	Beyotime	Cat: C3018S	
**Plasmid**			
HA-P62	Yubo Biotechnology, China		
HA-K13R	Yubo Biotechnology, China		
HA-K435R	Yubo Biotechnology, China		
HA-K165R	Yubo Biotechnology, China		
HA-K165Q	Yubo Biotechnology, China		
Flag-HDAC10	Yubo Biotechnology, China		
Flag-Vector	Yubo Biotechnology, China		
**Software and Algorithms**			
GraphPad Prism 8.0	https://www.graphpad.com		N/A
Image J	https://imagej.net/Welcome		N/A
Real-Time PCR Systems	Applied Biosystem		N/A
CSS-Palm 3.0	csspalm.biocuckoo.org		
**Mice**			
C57BL/6 wild-type (WT) mice	GemPharmatech Co., Ltd., China		N/A
*Hdac10*^*fl/fl*^ mice	GemPharmatech Co., Ltd., China		N/A

#### Ethical approval

All animal experiments were approved by the Animal Ethics Committee of Guangdong Medical University.

### Statistical analysis

Results were represented as mean ±SEM. All datas were analyzed by GraphPad Prism 8.0 (San Diego, CA). Unpaired t-tests were used to compare differences between two experimental groups. One-Way ANOVA followed by Tukey's multiple comparisons test was used for multiple group comparisons. *p* < 0.05 was considered meaningful.

### Supplementary Information

Additional file 1: Supplementary Fig. S1. HDAC10 expression is increased in the macrophage cells following LPS exposure. (A, B) Immunofluorescence analysis demonstrating the expression of HDAC10 (green) after 12 h of LPS challenge in MH-S cells. (C, D) HDAC10 protein expression in BMDMs after 100 μg/ml LPS stimulation for 0-12 h and at different concentrations for 12 h. (E, F) HDAC10 mRNA level in BMDMs after 100 μg/ml LPS stimulation for 0-12 h and at different concentrations for 12 h. The data are presented as the mean ±SEM and represent three independent experiments (B, E, F). ^*^p < 0.05, ^**^p < 0.01, ^***^p < 0.001, and ^****^p < 0.0001. 

Additional file 2: Supplementary Fig. S2. *Hdac10-*deficient BMDMs inhibit cytokine secretion when exposed to LPS. (A, B) The protein and mRNA levels of HDAC10 in BMDMs from *Hdac10*^*fl/fl*^ and *Hdac10*^*fl/fl*^*-LysMCre mice*. (C-F) The mRNA levels of *Cxcl1*, *Cxcl2*, *Il-6*, and *Tnf-α* in BMDMs after treated with LPS for 3 h. The data are presents as the mean ± SEM and represent three independent experiments (B-F). ^*^p < 0.05, ^**^p < 0.01, ^***^p< 0.001.

Additional file 3: Supplementary Fig. S3. *Hdac10* deficiency acetylates P62-K165 to reduce cytokine secretion. (A) The transiently transfection efficiency of WT-P62 or P62 mutant plasmids (K165R and K165Q) in *Hdac10*^*fl/fl*^*-LysMCre* BMDMs. (B-E) *Hdac10*^*fl/fl*^*-LysMCre* BMDMs transiently transfected with WT-P62 or P62 mutant plasmids (K165R and K165Q) for 48h, and then *Hdac10*^*fl/fl*^*-LysMCre* BMDMs were stimulation with LPS for 3 h. The mRNA levels of *Cxcl1, Cxcl2, Il-6* and *Tnf-α* in *Hdac10*^*fl/fl*^*-LysMCre* BMDMs detected by qRT-PCR. The data are presents as the mean ± SEM and represent three independent experiments (B-F). ^*^p < 0.05, ^**^p < 0.01, ^***^p < 0.001.

Additional file 4: Supplementary Fig. S4. HDAC10 inhibitor treatment attenuates inflammatory cytokine production in MH-S cells after LPS stimulation. (A) The protein level of P62 in MH-S cells after treated with LPS and SAB. (B-E) The mRNA levels of *Cxcl1*, *Cxcl2*, *Il-6*, and *Tnf-α*. The data are presents as the mean ± SEM and represent three independent experiments(B-E). ^*^p < 0.05, ^**^p < 0.01, ^***^p < 0.001, and ^****^p< 0.0001.

Additional file 5: Supplementary Fig. S5. Schematic diagram for Myeloid-specific *Hdac10* deletion protects against LPS-induced acute lung injury via P62 acetylation at lysine 165.

Additional file 6.

Additional file 7.

## Data Availability

No datasets were generated or analysed during the current study.

## Abbreviations

HDAC10: Histone deacetylase 10
BMDMs: Primary bone marrow-derived macrophages
SAB: Salvianolic acid B
LPS: Lipopolysaccharide

## References

[1] Matuschak GM, Lechner AJ (2010). Acute lung injury and the acute respiratory distress syndrome: pathophysiology and treatment. Mo Med..

[2] Mokra D (2020). Acute lung injury - from pathophysiology to treatment. Physiol Res..

[3] Cheng P, Li S, Chen H (2021). Macrophages in Lung Injury, Repair, and Fibrosis. Cells..

[4] Chen X, Tang J, Shuai W, Meng J, Feng J, Han Z (2020). Macrophage polarization and its role in the pathogenesis of acute lung injury/acute respiratory distress syndrome. Inflamm Res..

[5] Xia L, Zhang C, Lv N, Liang Z, Ma T, Cheng H (2022). AdMSC-derived exosomes alleviate acute lung injury via transferring mitochondrial component to improve homeostasis of alveolar macrophages. Theranostics..

[6] Sun S, Yao Y, Huang C, Xu H, Zhao Y, Wang Y (2022). CD36 regulates LPS-induced acute lung injury by promoting macrophages M1 polarization. Cell Immunol..

[7] Shvedunova M, Akhtar A (2022). Modulation of cellular processes by histone and non-histone protein acetylation. Nat Rev Mol Cell Biol..

[8] Xu C, Tang J, Yang Q, Zhao H, Liu Y, Cao J (2021). Histone deacetylase 5 deacetylates the phosphatase PP2A for positively regulating NF-kappaB signaling. J Biol Chem..

[9] Youn GS, Lee KW, Choi SY, Park J (2016). Overexpression of HDAC6 induces pro-inflammatory responses by regulating ROS-MAPK-NF-κB/AP-1 signaling pathways in macrophages. Free Radic Biol Med..

[10] Liao W, Sun J, Liu W, Li W, Jia J, Ou F (2019). HDAC10 upregulation contributes to interleukin 1β-mediated inflammatory activation of synovium-derived mesenchymal stem cells in temporomandibular joint. J Cell Physiol..

[11] Wang K, Chen Y, Zhang P, Lin P, Xie N, Wu M (2019). Protective Features of Autophagy in Pulmonary Infection and Inflammatory Diseases. Cells..

[12] Liao SX, Sun PP, Gu YH, Rao XM, Zhang LY, Ou-Yang Y (2019). Autophagy and pulmonary disease. Ther Adv Respir Dis..

[13] Hennig P, Fenini G, Di Filippo M, Karakaya T, Beer HD (2021). The Pathways Underlying the Multiple Roles of p62 in Inflammation and Cancer. Biomedicines..

[14] Kong L, Deng J, Zhou X, Cai B, Zhang B, Chen X (2021). Sitagliptin activates the p62-Keap1-Nrf2 signalling pathway to alleviate oxidative stress and excessive autophagy in severe acute pancreatitis-related acute lung injury. Cell Death Dis..

[15] Li T, Wu YN, Wang H, Ma JY, Zhai SS, Duan J (2020). Dapk1 improves inflammation, oxidative stress and autophagy in LPS-induced acute lung injury via p38MAPK/NF-κB signaling pathway. Mol Immunol..

[16] Rubenfeld GD, Caldwell E, Peabody E, Weaver J, Martin DP, Neff M (2005). Incidence and outcomes of acute lung injury. N Engl J Med..

[17] Hai R, Yang D, Zheng F, Wang W, Han X, Bode AM (2022). The emerging roles of HDACs and their therapeutic implications in cancer. Eur J Pharmacol..

[18] Cheng F, Zheng B, Wang J, Zhao G, Yao Z, Niu Z (2021). Histone deacetylase 10, a potential epigenetic target for therapy. Biosci Rep..

[19] Dahiya S, Beier UH, Wang L, Han R, Jiao J, Akimova T (2020). HDAC10 deletion promotes Foxp3(+) T-regulatory cell function. Sci Rep..

[20] Yang Y, Huang Y, Wang Z, Wang HT, Duan B, Ye D (2016). HDAC10 promotes lung cancer proliferation via AKT phosphorylation. Oncotarget..

[21] Wang Y, Huang Y, Liu J, Zhang J, Xu M, You Z (2020). Acetyltransferase GCN5 regulates autophagy and lysosome biogenesis by targeting TFEB. EMBO Rep..

[22] Z WK, W JM, W X, W BJ, Z ZH, F J (2022). Degradation of HDAC10 by autophagy promotes IRF3-mediated antiviral innate immune responses.. Sci Signal..

[23] Shutong L, Yu J, Jia W, Huafei D, Shifan Y, Huili W (2022). HO-1/autophagic flux axis alleviated sepsis-induced acute lung injury via inhibiting NLRP3 inflammasome. Cell Signal..

