# Supplementary figures and images for: Myeloid-specific Hdac10 deletion protects against LPS-induced acute lung injury via P62 acetylation at lysine 165
Source: Respir Res. 2024 Jul 2;25:263. doi: 10.1186/s12931-024-02891-2 (PMC11221109; doi:10.1186/s12931-024-02891-2)

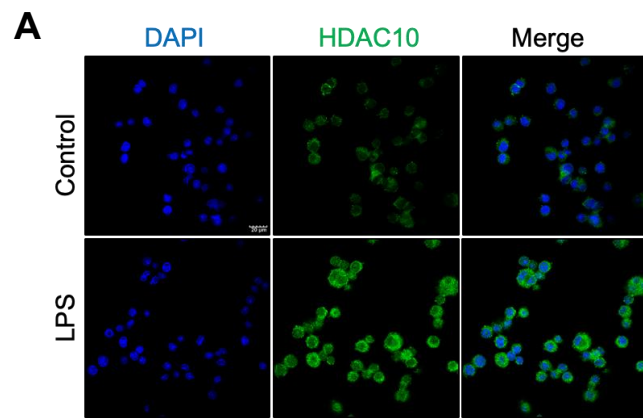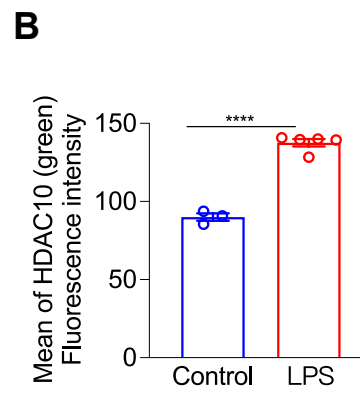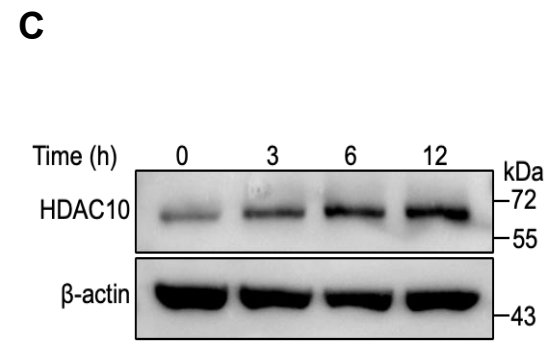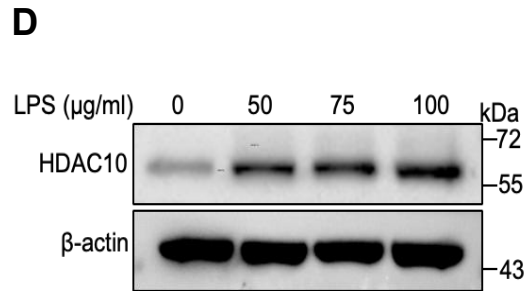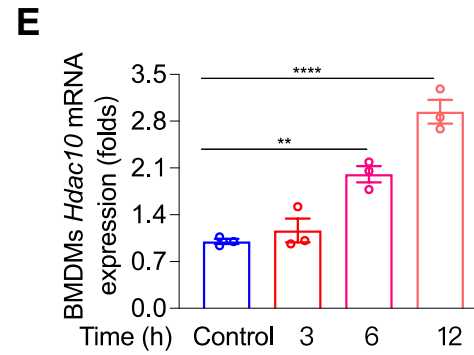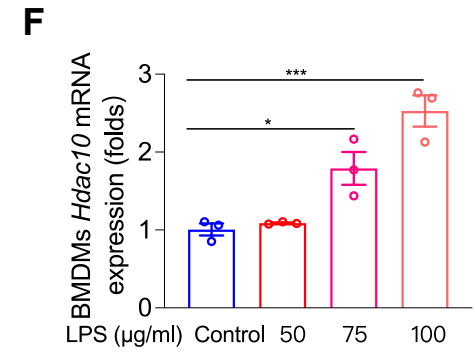

Supplement: Supplementary file 1 — Additional file 1: Supplementary Fig. S1. HDAC10 expression is increased in the macrophage cells following LPS exposure. (A, B) Immunofluorescence analysis demonstrating the expression of HDAC10 (green) after 12 h of LPS challenge in MH-S cells. (C, D) HDAC10 protein expression in BMDMs after 100 μg/ml LPS stimulation for 0-12 h and at different concentrations for 12 h. (E, F) HDAC10 mRNA level in BMDMs after 100 μg/ml LPS stimulation for 0-12 h and at different concentrations for 12 h. The data are presented as the mean ±SEM and represent three independent experiments (B, E, F). *p < 0.05, **p < 0.01, ***p < 0.001, and ****p < 0.0001. [file 12931_2024_2891_MOESM1_ESM.pdf]

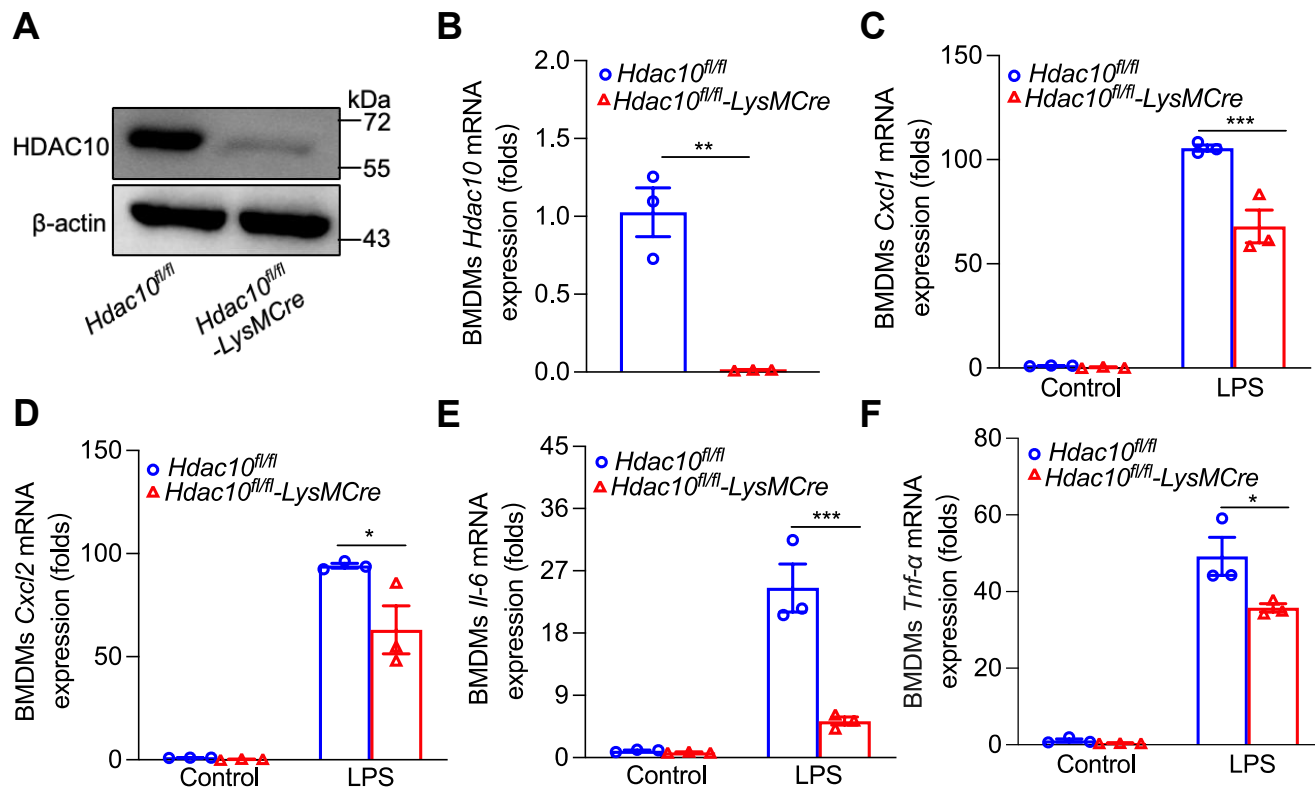

Supplement: Supplementary file 2 — Additional file 2: Supplementary Fig. S2. Hdac10-deficient BMDMs inhibit cytokine secretion when exposed to LPS. (A, B) The protein and mRNA levels of HDAC10 in BMDMs from Hdac10fl/fl and Hdac10fl/fl-LysMCre mice. (C-F) The mRNA levels of Cxcl1, Cxcl2, Il-6, and Tnf-α in BMDMs after treated with LPS for 3 h. The data are presents as the mean ± SEM and represent three independent experiments (B-F). *p < 0.05, **p < 0.01, ***p< 0.001. [file 12931_2024_2891_MOESM2_ESM.pdf]

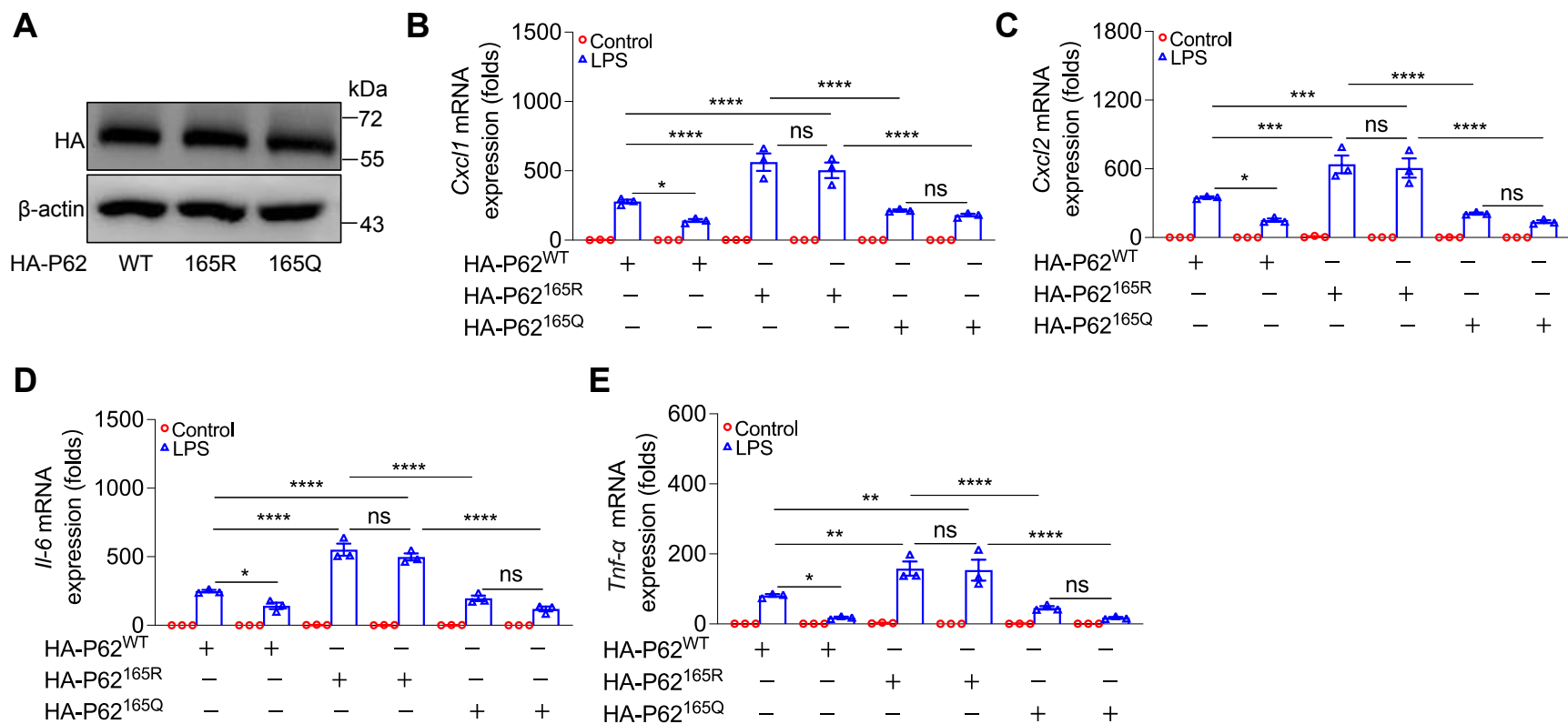

Supplement: Supplementary file 3 — Additional file 3: Supplementary Fig. S3. Hdac10 deficiency acetylates P62-K165 to reduce cytokine secretion. (A) The transiently transfection efficiency of WT-P62 or P62 mutant plasmids (K165R and K165Q) in Hdac10fl/fl-LysMCre BMDMs. (B-E) Hdac10fl/fl-LysMCre BMDMs transiently transfected with WT-P62 or P62 mutant plasmids (K165R and K165Q) for 48h, and then Hdac10fl/fl-LysMCre BMDMs were stimulation with LPS for 3 h. The mRNA levels of Cxcl1, Cxcl2, Il-6 and Tnf-α in Hdac10fl/fl-LysMCre BMDMs detected by qRT-PCR. The data are presents as the mean ± SEM and represent three independent experiments (B-F). *p < 0.05, **p < 0.01, ***p < 0.001. [file 12931_2024_2891_MOESM3_ESM.pdf]

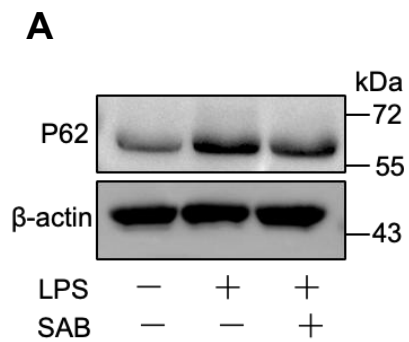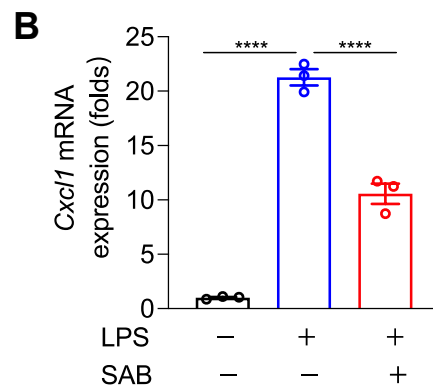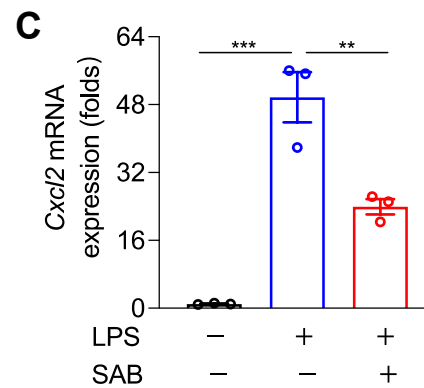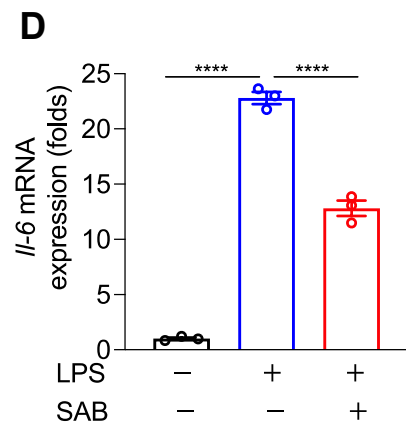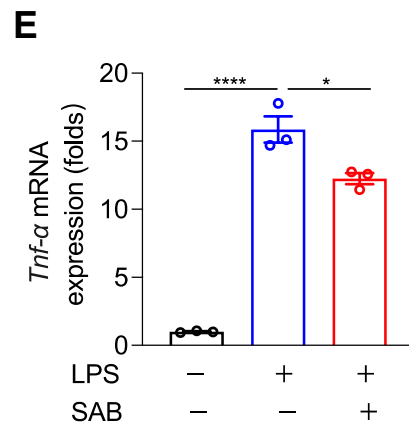

Supplement: Supplementary file 4 — Additional file 4: Supplementary Fig. S4. HDAC10 inhibitor treatment attenuates inflammatory cytokine production in MH-S cells after LPS stimulation. (A) The protein level of P62 in MH-S cells after treated with LPS and SAB. (B-E) The mRNA levels of Cxcl1, Cxcl2, Il-6, and Tnf-α. The data are presents as the mean ± SEM and represent three independent experiments(B-E). *p < 0.05, **p < 0.01, ***p < 0.001, and ****p< 0.0001. [file 12931_2024_2891_MOESM4_ESM.pdf]

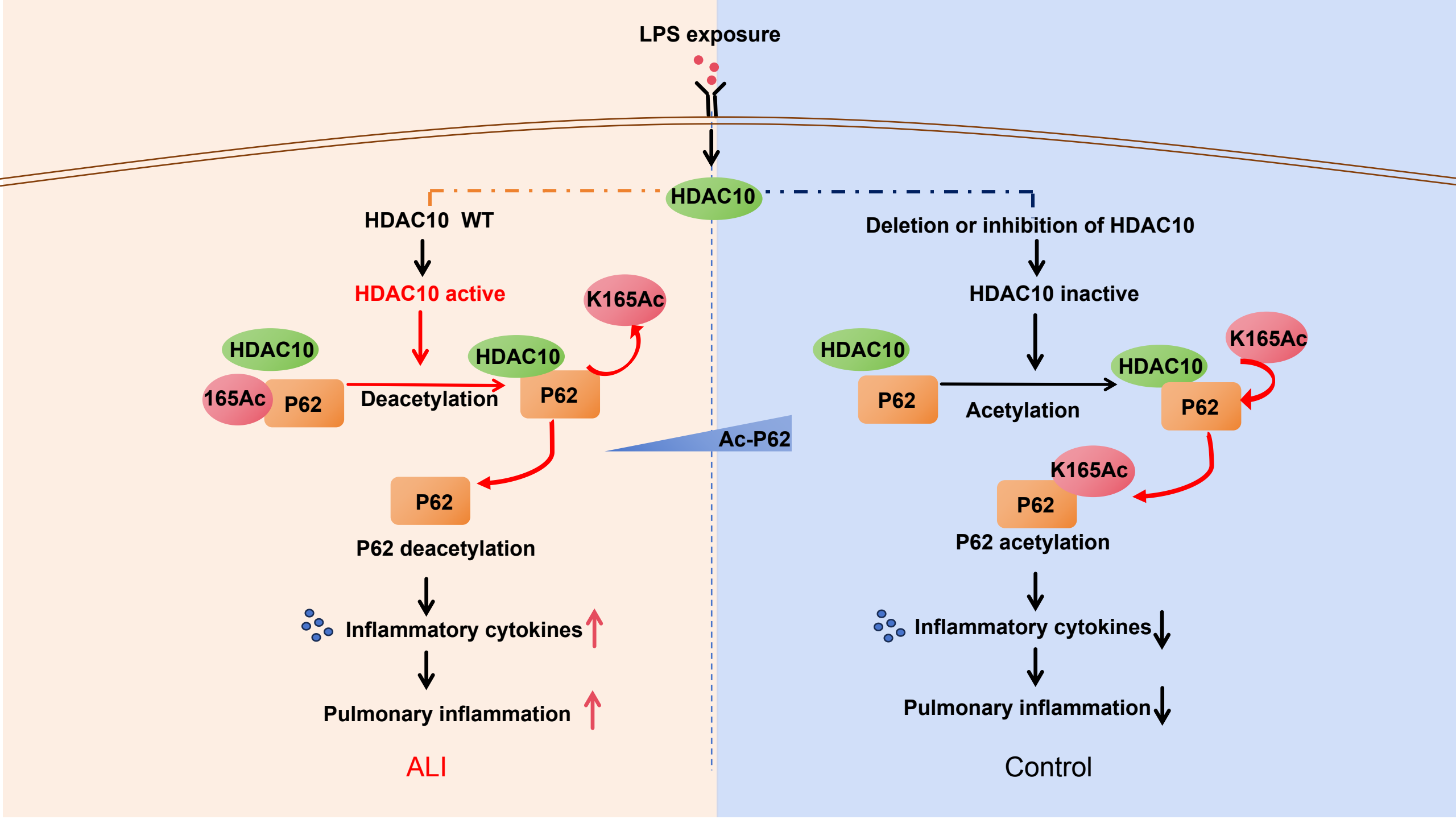

Supplement: Supplementary file 5 — Additional file 5: Supplementary Fig. S5. Schematic diagram for Myeloid-specific Hdac10 deletion protects against LPS-induced acute lung injury via P62 acetylation at lysine 165. [file 12931_2024_2891_MOESM5_ESM.pdf]
