# Supplementary material for: Myeloid-specific Hdac10 deletion protects against LPS-induced acute lung injury via P62 acetylation at lysine 165
Source: Respir Res. 2024 Jul 2;25:263. doi: 10.1186/s12931-024-02891-2 (PMC11221109; doi:10.1186/s12931-024-02891-2)

Fig. 1B

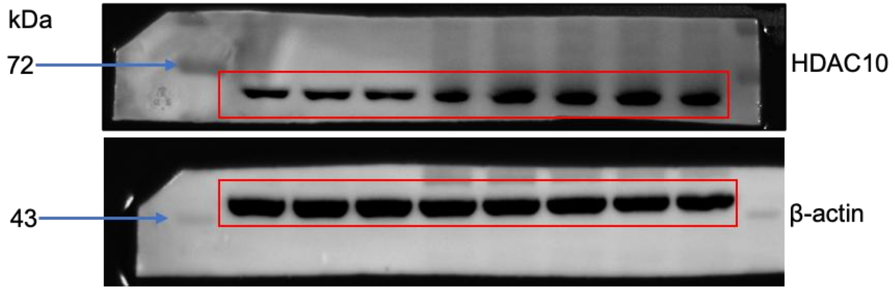

Fig. 1H

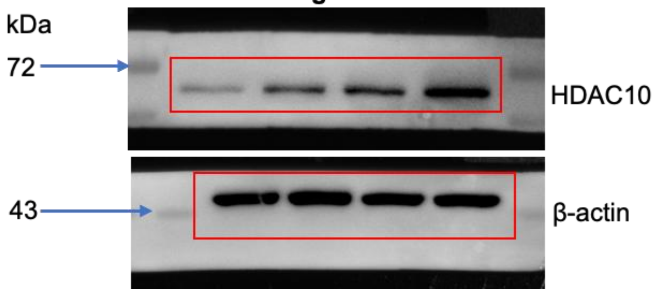

Fig. 1I

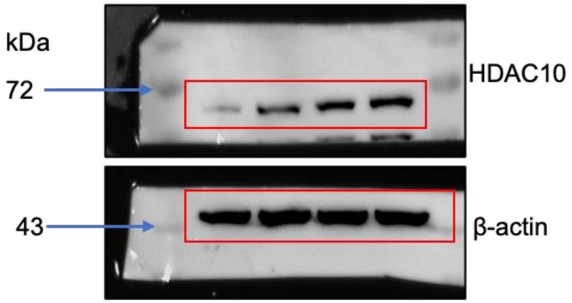

Fig. 3A

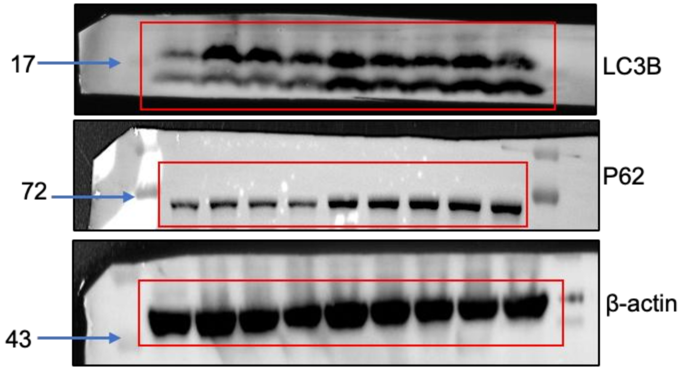

Fig. 3B

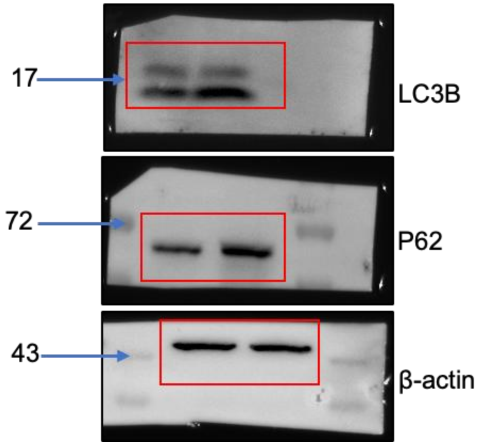

Fig. 3C

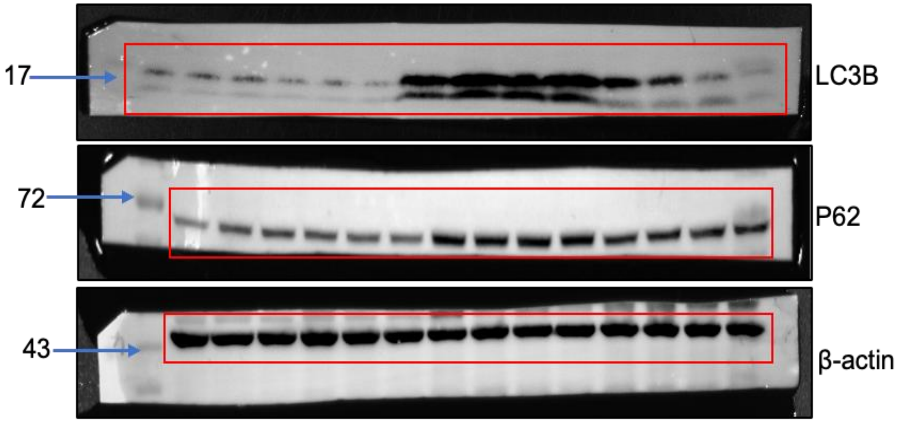

Fig. 3E

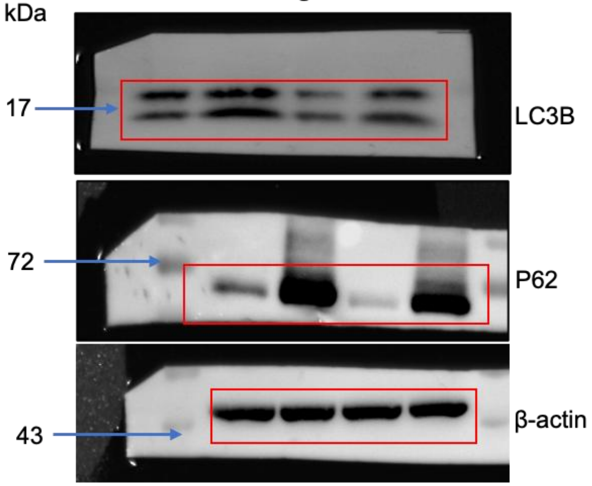

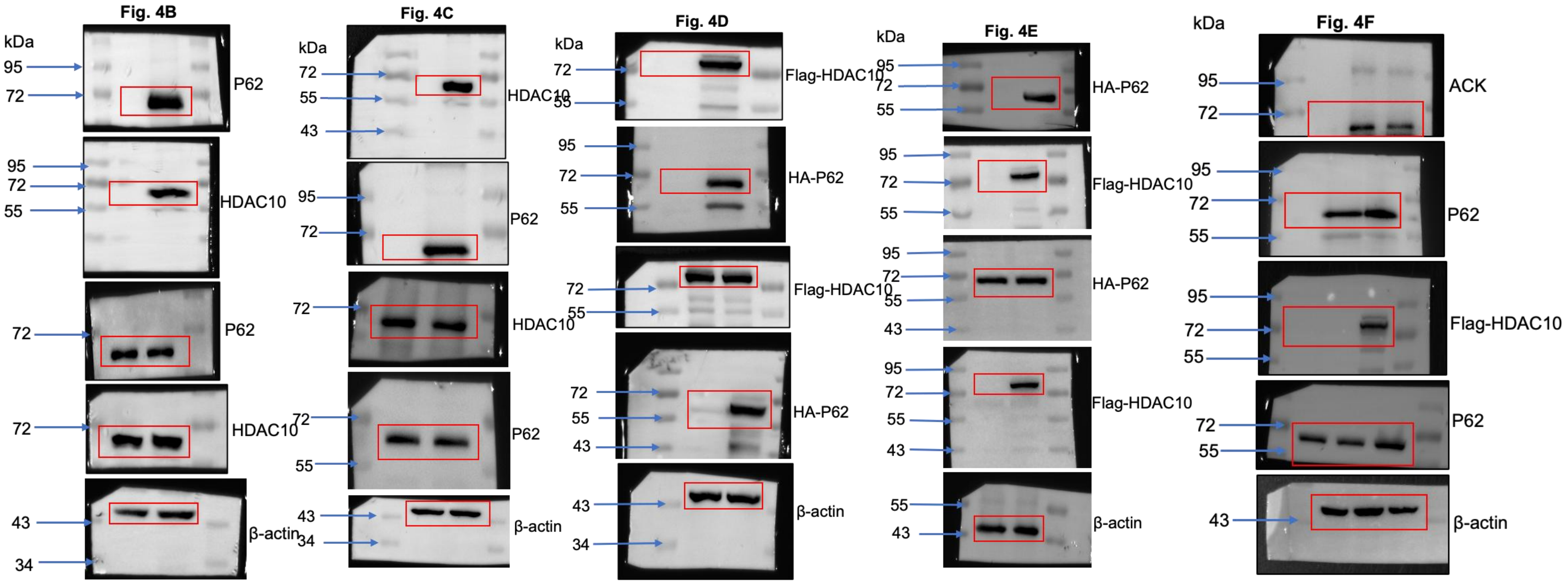

Fig. 4B
kDa
95
72
P62
95
72
55
HDAC10
72
P62
72
HDAC10
43
34
β-actin
Fig. 4C
kDa
72
55
43
HDAC10
95
72
P62
72
HDAC10
72
55
P62
43
34
β-actin
Fig. 4D
kDa
72
55
Flag-HDAC10
95
72
55
HA-P62
72
55
Flag-HDAC10
72
55
43
HA-P62
43
34
β-actin
Fig. 4E
kDa
95
72
55
HA-P62
95
72
55
Flag-HDAC10
95
72
55
43
HA-P62
95
72
55
43
Flag-HDAC10
55
43
β-actin
Fig. 4F
kDa
95
72
ACK
95
72
55
P62
95
72
55
Flag-HDAC10
72
55
P62
43
β-actin

Fig. 4G

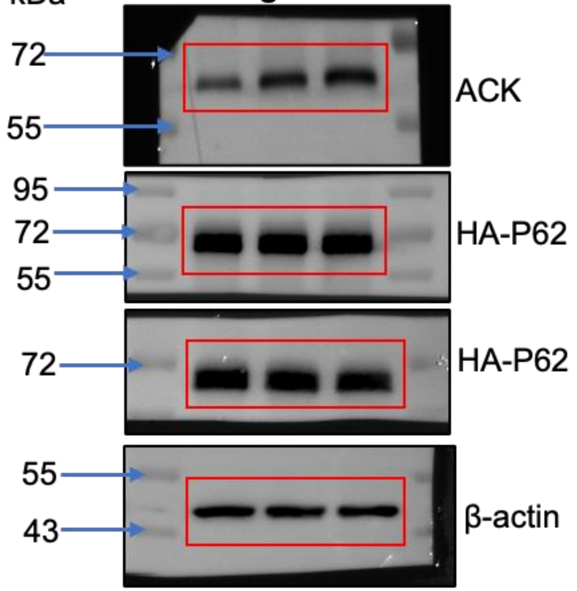

Fig. 4I

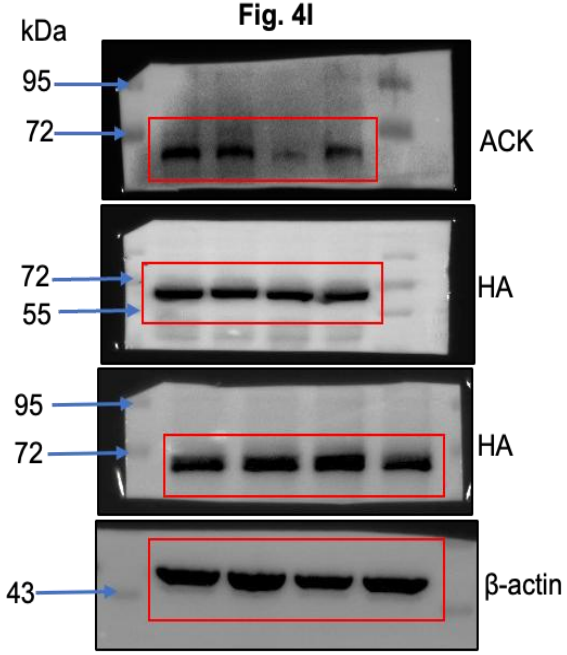

Fig. 4J

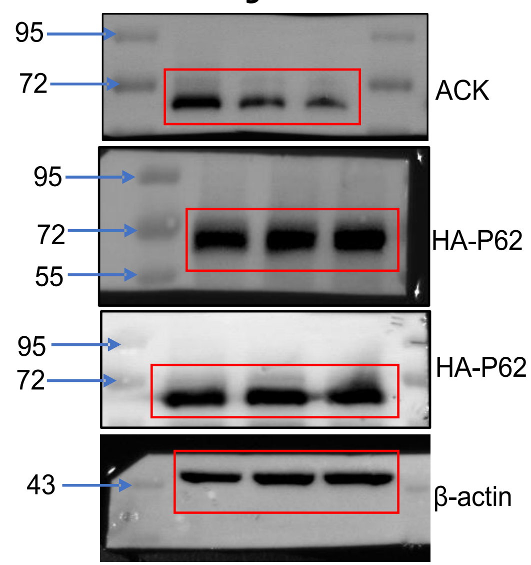

Fig. 4K

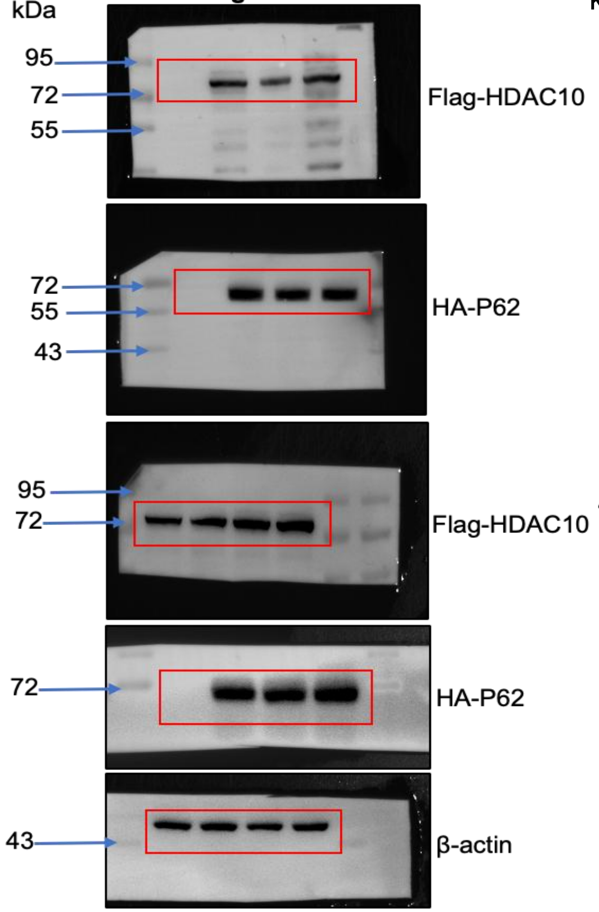

Fig. 5A

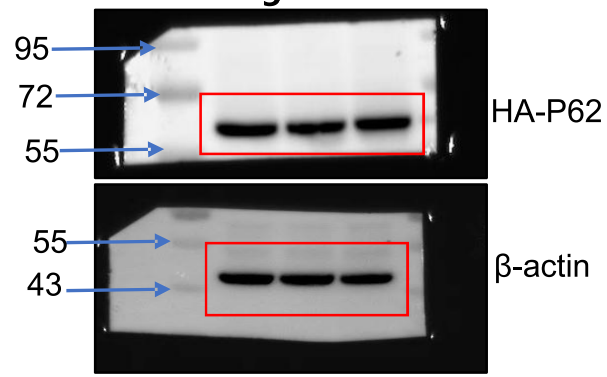

Fig. 5F

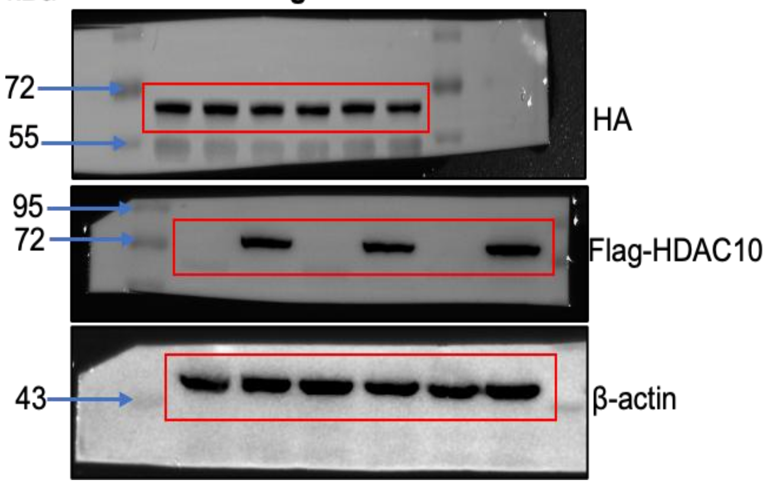

Fig. 6E

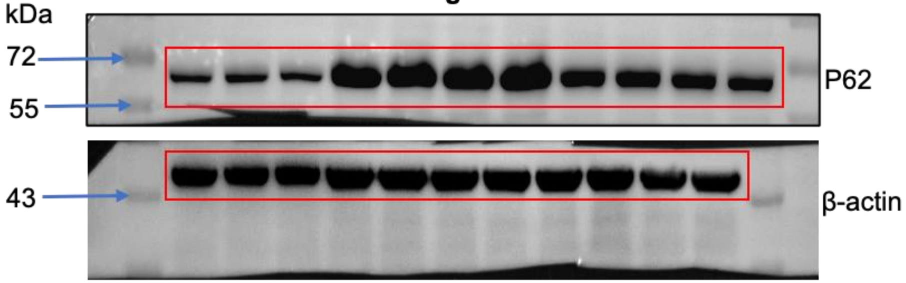

Fig. 6F

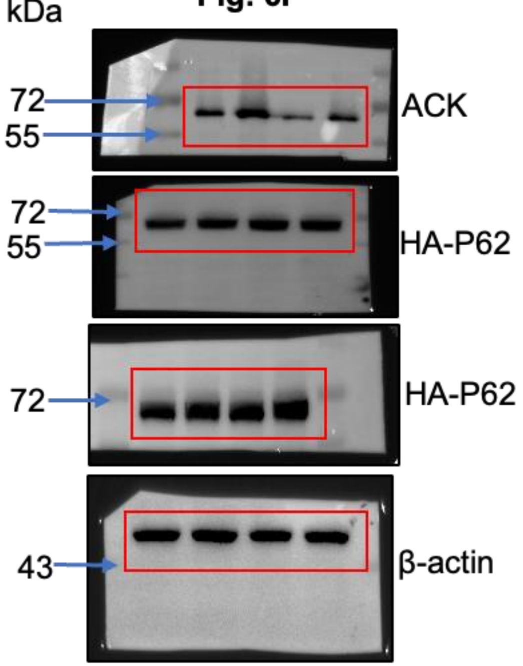

Supplementary Fig. S1C

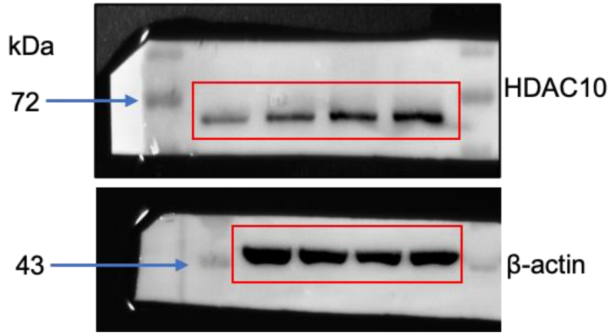

Supplementary Fig. S1D

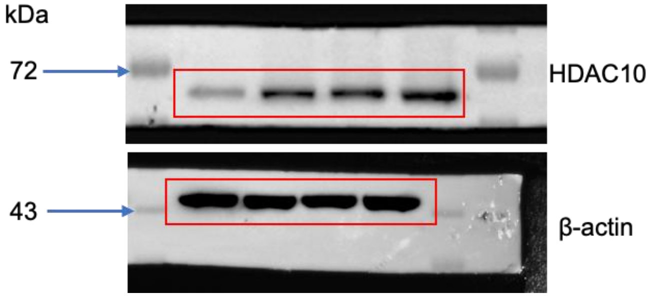

Supplementary Fig. S2A

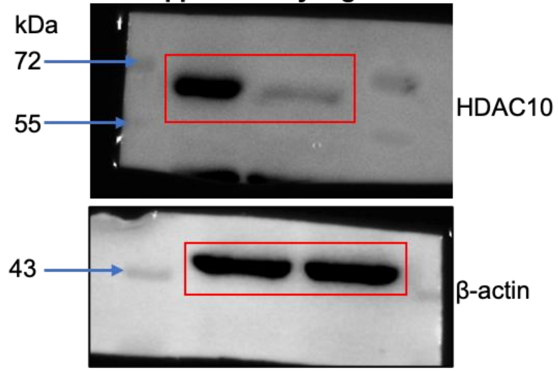

Supplementary Fig. S3A

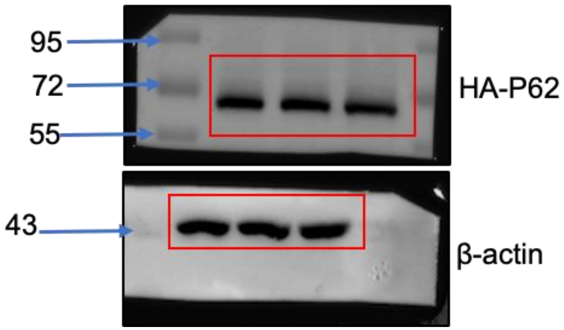

Supplementary Fig. S4A

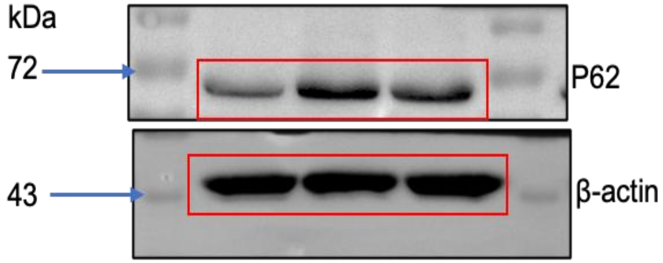

Supplement: Supplementary file 7 — Additional file 7. [file 12931_2024_2891_MOESM7_ESM.pdf]
